# Role of cellular factors in the replication of human retroviruses: recent insights

**DOI:** 10.1186/1742-4690-8-S2-O21

**Published:** 2011-10-03

**Authors:** Kuan-Teh Jeang

**Affiliations:** 1 National Institutes of Health, Bethesda, MD 20892, USA

## 

We and others have described the use of genome-wide screenings for cellular factors that contribute to HIV-1 replication [1,2]. One aspect of the HIV-1 life cycle important to viral replication is the post-transcriptional regulation of HIV-1 gene expression. In HIV-1 biology, a key step in gene expression is the post-transcriptional export of intron-containing viral RNAs which bypass the normal mechanism(s) that retain cellular intron-containing RNAs in the nucleus. Specific signals on the viral RNAs, such as instability sequences (INS) and Rev responsive element (RRE), are binding sites for viral and cellular factors that serve to regulate RNA-export. The HIV-1 encoded viral Rev protein binds to the RRE found on unspliced and incompletely spliced viral RNAs. Binding by Rev directs the export of these RNAs from the nucleus to the cytoplasm. Previously, Rev co-factors have been found to include cellular factors such as CRM1, DDX3, PIMT and Matrin3 [3,4]. I will discuss these and other cellular factors that influence HIV-1 replication.

## References

[B1] Yeung ML Houzet L Yedavalli VS Jeang KT A Genome-wide Short Hairpin RNA Screening of Jurkat T-cells for Human Proteins Contributing to Productive HIV-1 Replication J Biol Chem 2009 284 19463 73 10.1074/jbc.M109.010033 19460752 PMC2740572

[B2] Kok KH Lei T Jin DY siRNA and shRNA screens advance key understanding of host factors required for HIV-1 replication Retrovirology 2009 6 78 10.1186/1742-4690-6-78 19712452 PMC2743632

[B3] Yedavalli VSRK Jeang KT Matrin 3 is a co-factor for HIV-1 Rev in regulating post-transcriptional viral gene expression Retrovirology 2011 8 61 10.1186/1742-4690-8-61 21771347 PMC3160905

[B4] Kula A Guerra J Knezevich A Kleva D Myers MP Marcello A Characterization of the HIV-1 RNA associated proteome identifies Matrin 3 as a nuclear cofactor of Rev function Retrovirology 2011 8 60 10.1186/1742-4690-8-60 21771346 PMC3160904

